# Early detection of subclinical left ventricular dysfunction after breast cancer radiation therapy using speckle-tracking echocardiography: association between cardiac exposure and longitudinal strain reduction (BACCARAT study)

**DOI:** 10.1186/s13014-019-1408-8

**Published:** 2019-11-14

**Authors:** Valentin Walker, Olivier Lairez, Olivier Fondard, Atul Pathak, Baptiste Pinel, Christian Chevelle, Denis Franck, Gaëlle Jimenez, Jérémy Camilleri, Loïc Panh, David Broggio, Sylvie Derreumaux, Marie-Odile Bernier, Dominique Laurier, Jean Ferrières, Sophie Jacob

**Affiliations:** 1 Laboratory of Epidemiology (LEPID), PSE-SANTE, SESANE, Institute for Radiological Protection and Nuclear Safety (IRSN), Fontenay-aux-Roses, France; 2 Cardiac Imaging Center, Toulouse University Hospital, Toulouse, France; 3 Department of Cardiology, Clinique Pasteur, Toulouse, France; 4 Department of Radiation Oncology (Oncorad), Clinique Pasteur, Toulouse, France; 5 Department of Cardiac Arrhythmia, Clinique Pasteur, Toulouse, France; 6 Department of Dosimetry, Institute for Radiological Protection and Nuclear Safety (IRSN), Fontenay-aux-Roses, France; 7 Institute for Radiological Protection and Nuclear Safety (IRSN), Fontenay-aux-Roses, France; 8 Department of Cardiology, Toulouse University Hospital, Toulouse, France; 9 INSERM, UMR1027, Toulouse, France

**Keywords:** Breast Cancer, 3D conformal radiation therapy, Cardiac dysfunction, Strain imaging, Cardiac Dosimetry

## Abstract

**Background:**

Breast cancer (BC) radiotherapy (RT) can induce cardiotoxicity, with adverse events often observed many years after BC RT. Subclinical left ventricular (LV) dysfunction can be detected early after BC RT with global longitudinal strain (GLS) measurement based on 2D speckle-tracking echocardiography. This 6-month follow-up analysis from the BACCARAT prospective study aimed to investigate the association between cardiac radiation doses and subclinical LV dysfunction based on GLS reduction.

**Methods:**

The patient study group consisted of 79 BC patients (64 left-sided BC, 15 right-sided BC) treated with RT without chemotherapy. Echocardiographic parameters, including GLS, were measured before RT and 6 months post-RT. The association between subclinical LV dysfunction, defined as GLS reduction > 10%, and radiation doses to whole heart and the LV were performed based on logistic regressions. Non-radiation factors associated with subclinical LV dysfunction including age, BMI, hypertension, hypercholesterolemia and endocrine therapy were considered for multivariate analyses.

**Results:**

A mean decrease of 6% in GLS was observed (− 15.1% ± 3.2% at 6 months vs. − 16.1% ± 2.7% before RT, *p* = 0.01). For left-sided patients, mean heart and LV doses were 3.1 ± 1.3 Gy and 6.7 ± 3.4 Gy respectively. For right-sided patients, mean heart dose was 0.7 ± 0.5 Gy and median LV dose was 0.1 Gy. Associations between GLS reduction > 10% (37 patients) and mean doses to the heart and the LV as well as the V20 were observed in univariate analysis (Odds Ratio = 1.37[1.01–1.86], *p* = 0.04 for Dmean Heart; OR = 1.14 [1.01–1.28], *p* = 0.03 for Dmean LV; OR = 1.08 [1.01–1.14], *p* = 0.02 for LV V20). In multivariate analysis, these associations did not remain significant after adjustment for non-radiation factors. Further exploratory analysis allowed identifying a subgroup of patients (LV V20 > 15%) for whom a significant association with subclinical LV dysfunction was found (adjusted OR = 3.97 [1.01–15.70], *p* = 0.048).

**Conclusions:**

This analysis indicated that subclinical LV dysfunction defined as a GLS decrease > 10% is associated with cardiac doses, but adjustment for non-radiation factors such as endocrine therapy lead to no longer statistically significant relationships. However, LV dosimetry may be promising to identify high-risk subpopulations. Larger and longer follow-up studies are required to further investigate these associations.

**Trial registration:**

ClinicalTrials.gov: NCT02605512, Registered 6 November 2015 - Retrospectively registered

## Background

Breast cancer (BC) radiotherapy (RT) reduces BC recurrence and improves survival [1]. However, increased risk of cardiac death many years after BC RT has been documented [2]. For long-term radiotherapy-induced cardiac complications, dose-response relationships between the mean heart dose and the rate of major coronary events were observed [3–5]. Long before the onset of clinically relevant cardiac events, evaluation of early myocardial dysfunction was investigated after BC RT [6]. This was based on two-dimensional speckle tracking echocardiography (2DSTE) that has allowed accurate measurements of global and regional myocardial deformation with strain [7, 8]. Several studies have showed the higher sensitivity and prognostic value of the global longitudinal strain (GLS), compared with left ventricular ejection fraction (LVEF), for early detection of left ventricular dysfunction, and it has been shown that detecting a decreased LVEF after RT may be too late for treatment [9, 10].

In most of previous studies on early myocardial dysfunction post BC RT, a statistically significant decrease in GLS was observed among left-sided BC patients, ranging from 5 to 14% at different post-RT time points from few weeks to 14 months [11–16], whereas no measurable alteration of LVEF was observed. However, in these studies, no or few results were specifically presented for patients with a drop in GLS > 10% whereas this threshold is considered to define subclinical left ventricular dysfunction and has been reported to be predictive of subsequent cardiotoxicity [17, 18].

Knowledge on the relationship between cardiac exposure and the decrease of strain is limited. A dose-related regional myocardial dysfunction in the acute phase after RT was found in left-sided BC patients with the greatest reduction in the apical part of the left ventricle, which received the highest radiation dose [15], but no significant association was found between the mean heart dose and the GLS reduction [19]. Further studies are needed to investigate the association between cardiac exposure and the evolution of GLS after RT, considering in particular the doses absorbed to the whole heart as well as to cardiac substructures such as the left ventricle or the coronary arteries. Indeed, these doses could enhance knowledge on the dose-response relationship according to the type of cardiotoxicity and its location [20].

Based on the BACCARAT prospective cohort of BC patients treated with 3D-CRT (3-Dimensional Conformal Radiotherapy), we aimed to present a 6-month follow-up analysis of the association between radiation exposure to the whole heart and the left ventricle (LV) and the evolution of GLS from baseline to 6 months after RT, in particular for subclinical LV dysfunction defined as GLS reduction > 10%, a secondary endpoint of BACCARAT.

## Materials and methods

### Patient population

This prospective, monocentric observational clinical study [21] initially included 118 female patients of the Clinic Pasteur Toulouse from October 2015 to December 2017, aged 40 to 75 years old, mainly with left unilateral BC, and in a smaller proportion with right-sided unilateral BC, followed from baseline before RT to 6 months after RT. All patients were treated with adjuvant 3D-CRT after breast conserving surgery or mastectomy, without chemotherapy. Five patients withdrew consent and 8 patients had abnormal LVEF before RT (LVEF < 45%). For this analysis, we excluded all echocardiographies for which the image quality was too low for a reliable assessment of longitudinal strain (*n* = 20). In addition, 6 patients without available cardiac dosimetry (see details below) were excluded. Finally, the patient study group consisted of 79 patients.

### Radiotherapy treatment

After the surgical treatment of BC, all patients were treated with 3D-CRT with 6 and 25 MV photon beams by tangential fields. The planning target volume dose was 50 Gy delivered in 25 daily fractions of 2 Gy over 5 weeks or 47 Gy delivered in 20 daily fractions of 2.35 Gy over 5 weeks for patients treated between January 2016 and May 2016 (technical problems arose in one 3D-CRT machine during this period and this hypofractionation choice was only driven by the need to slightly limit the number of sessions per patient). For most patients, 6MV photons were used, except few cases of patients with big breast where 25 MV additional photons were used. Additional boost of 9–15 Gy could be applied to the tumor site using photon/electron beams with energies ranging from 6 MeV to 18 MeV. The treatment planning system (TPS) used to perform dose calculations was Eclipse™ with the Analytical Anisotropic Algorithm (AAA v13.6) (Varian Medical System, Palo Alto, CA, USA). Each patient’s RT was planned such that the dose distribution was optimized and normalized to the International Commission on Radiation Units and Measurements (ICRU) reference point of the breast and to achieve QUANTEC dose constraints to organs at risk including the heart [22].

### Evaluation of radiation doses

The methodology of the complete evaluation of radiation doses in BACCARAT patients was previously described [20, 21]. Only whole heart and left ventricle were considered in the analyses presented here. Dose-Volume-Histogram (DVH) for the heart was generated by the Clinic Pasteur RT department. Manual delineation of the left ventricle was performed. Using the 3D dose matrix generated during planning treatment and the new delineated substructure, DVH for LV was generated with ISOGray TPS by the dosimetric department of IRSN in collaboration with the Clinic Pasteur RT department. From the DVHs, the following absorbed dose metrics for whole heart and left ventricle were calculated: Dmean (in Gy) is the volume-weighted mean dose; D2 (in Gy) is the minimal dose received by the most irradiated 2% of the structure volume, which can be considered as the near maximum dose; V20 (in %) is the relative volume exposed to at least 20 Gy.

### Echocardiographic examinations

A comprehensive 2D echocardiography study was performed at baseline before RT and 6 months after RT with a commercially available ultrasound Acuson S2000 (Siemens Medical Solutions USA, Inc. Malvern, USA), using a 3 MHz transducer. Image analysis was independently performed by a single blinded observer unaware of clinical data. LV ejection fraction (LVEF) was measured using the biplane Simpson’s method from apical two- and four-chamber windows. Myocardial function by longitudinal myocardial strain was calculated using two-dimensional speckle tracking echocardiography (2DSTE) and the automated function imaging technique for tracking of acoustic markers (speckles) [23]. The 2DSTE vendor offline Syngo Velocity Vector Imaging 2.0 software (Siemens Medical Solutions USA, Inc., Moutain View, Calif, USA) was used. Images were analyzed in a 16-segment model according to the American Society of Echocardiography guidelines [24]. Moreover, the LV wall is not homogenous and includes an endocardial, a mid-myocardial, and an epicardial layer [25]. There are concurrent definitions as a basis for GLS calculation using endocardial, midwall, epicardial or average deformation. Measures of midwall longitudinal strain have been shown in several studies to be robust and reproducible [24] and for this analysis, we considered on the mid-myocardial layer for strain and strain rate. All segmental values were averaged to Global Longitudinal Strain (GLS, in %) and Global Longitudinal Strain Rate (GLSR, in s^− 1^).

### Statistical analysis

Continuous variables are presented with mean and standard deviation or median and (interquartile) range values. Categorical values are presented with percentages. Student’s *t*-test or Wilcoxon non-parametric test was used to compare continuous variables, adapted to paired samples for the comparison of echocardiographic variables (LVEF, GLS, GLSR) before RT and 6 months after RT. Percent change in GLS was defined as the ratio of the difference between RT + 6 months measurement and baseline measurement. A more than 10% decrease in the GLS is generally considered clinically relevant [16–18] to detect subclinical left ventricular dysfunction. For whole heart and left ventricle, we considered the following dosimetric parameters: Dmean, D2 (in Gy), and V20 (in %). The continuous association between dose measures and GLS change was illustrated with scatter plots with a non-parametric smooth (Lowess method). Comparison of the dosimetric parameters between the group GLS reduction > 10% and GLS reduction ≤10% was performed. In order to take into account multiple testing in these comparisons, we applied the Holm–Bonferroni method, a step-down procedure performed after conducting the 6 comparison tests. We analyzed the associations between GLS reduction > 10% and radiation and non-radiation factors in univariate analysis based on logistic regressions which provided odds ratios (OR). Cardiac radiation factors included the laterality of BC, Dmean, D2 and V20 of the heart and the left ventricle. Non radiation factors included age, BMI, smoking status, hypertension, diabetes, hypercholesterolemia and endocrine therapy. We also tested the association with the prescribed RT fractionation. For multivariate analysis, we only considered non-radiation variables with *p*-value < 0.20 in univariate analysis. Finally, *p*-value < 0.05 was considered statistically significant. All statistical analysis was performed using SAS statistical software for Windows (Version 9.4 – SAS Institute, Cary, NC).

## Results

### Study population

A total of 79 BC patients (64 left-sided and 15 right-sided) were included in the analysis. Baseline patients’ characteristics are shown in Table 1. The mean age was 58 ± 9 years. Most patients (84%) were diagnosed with an invasive ductal carcinoma, underwent breast conserving surgery (97%) and received endocrine therapy (76%). The prescribed radiation dose was 50 Gy in 25 sessions for 75% of the population. Concerning cardiac risk factors, 24% of the patients had hypertension and 30% hypercholesterolemia. No significant differences in these characteristics were observed between left-sided and right-sided BC patients (data not shown).

**Table 1:** Baseline characteristics of the study population: demographic, tumor and treatment cardiovascular history

	All patients *N* = 79
Age in years, mean ± SD	58 ± 9
Left-sided BC patients	64 (81%)
Type of cancer, n (%)	
In situ	13 (16%)
Invasive	66 (84%)
Grade, n (%)	
1	33 (42%)
2	37 (47%)
3	9 (11%)
Surgery, n (%)	
Breast conserving	76 (96%)
Mastectomy	3 (4%)
RT protocol, n (%)	
Standard - 50 Gy	59 (75%)
Hypo-fractionated - 47 Gy	20 (25%)
Boost, n (%)	75 (95%)
Regional lymph node irradiation, n (%)	24 (30%)
Supraclavicular alone	2
Internal mammary alone	2
Both	19
Endocrine therapy, n (%)	60 (76%)
Anti-aromatase	34
Tamoxifen	26
Body mass index in kg/m^2^, mean ± SD	24.4 ± 4.1
Smoking, n (%)	
Never-smokers	42 (53%)
Former smokers	23 (29%)
Current smokers	14 (18%)
Systolic blood pressure, in mmHg, mean ± SD	119 ± 13
Diastolic blood pressure in mmHg, mean ± SD	74 ± 10
Hypertension, n (%)	19 (24%)
Diabetes, n (%)	5 (6%)
Hypercholesterolemia, n (%)	24 (30%)

*BC* Breast Cancer, *RT* Radiotherapy, *SD* Standard Deviation

#### Cardiac doses

Radiation doses received by the heart and the left ventricle are detailed in Table 2. For left-sided patients, mean heart dose and mean LV dose were respectively 3.05 ± 1.31 Gy and 6.68 ± 3.36 Gy. Doses were much lower among right-sided patients. Half of left-sided patients received doses higher than 20 Gy to a volume of LV > 10%.

**Table 2:** Radiation doses to the heart and the left ventricle

	Left-sided BC patients *N* = 64		Right-sided BC patients *N* = 15	
	Mean ± SD Median (Q1-Q3)	Range	Mean ± SD Median (Q1-Q3)	Range
Heart				
Dmean (Gy)	3.05 ± 1.31	0.87–6.37	0.65 ± 0.49	0.25–2.17
D2 (Gy)	28.60 ± 16.85	4.16–48.87	2.57 ± 1.16	1.11–5.40
V20 (%)	3 (1–6)	0–10	0	0
Left Ventricle				
Dmean (Gy)	6.68 ± 3.36	1.16–13.42	0.09 (0.08–0.12)	0.06–1.24
D2 (Gy)	36.28 ± 14.81	4.49–55.48	0.38 (0.29–0.45)	0.22–2.53
V20 (%)	11 (4–18)	0–26	0	0

*BC* Breast Cancer, *SD* Standard Deviation, Q1-Q3: Interquartile range; D2 (in Gy): Minimal dose received by the most irradiated 2% of the structure volume; Dmean: Mean dose to the structure; V20 (in %): Relative volume of the structure exposed to at least 20 Gy

#### Echocardiographic findings

The results of echocardiography are summarized in Table 3. Compared with baseline pre-RT, no significant decrease in either LVEF or GLSR was observed. However, the GLS was significantly lower 6 months after RT at least for left-sided patients (− 16.0 ± 2.6% at baseline vs. -15.0 ± 3.0% at RT + 6 months, *p* = 0.02) with a mean decrease of 6%. We observed subclinical LV dysfunction defined as GLS reduction > 10% in 37 patients corresponding to 48% of left-sided BC patients and 40% of right-sided BC patients. Moreover, 18 patients with a GLS increase > 10% were observed (27% among right-sided BC patients and 21% among left-sided BC patients).

**Table 3:** Echocardiographic measurements at baseline and 6 months after RT

	All BC patients *N* = 79	Left-sided BC patients *N* = 64	Right-sided BC patients *N* = 15
LVEF (in %)			
Before RT	62 ± 7	61 ± 7	64 ± 8
RT + 6 months	60 ± 9	60 ± 9	63 ± 8
*p-*value	0.07	0.09	0.51
GLS (in %)			
Before RT	-16.1 ± 2.7	−16.0 ± 2.6	−16.2 ± 2.8
RT + 6 months	−15.1 ± 3.2	−15.0 ± 3.0	− 15.2 ± 4.0
*p-*value	**0.01**	**0.02**	0.26
Patients with GLS reduction > 10% after RT	37 (47%)	31 (48%)	6 (40%)
GLSR (in s^−1^)			
q Before RT	−0.93 ± 0.14	−0.92 ± 0.15	−0.98 ± 0.11
RT + 6 months	−0.98 ± 0.21	−0.96 ± 0.16	− 1.06 ± 0.34
*p-*value	0.09	0.15	0.37

*LVEF* Left Ventricular Ejection Fraction, *GLS* Global Longitudinal Strain, *GLSR* Global Longitudinal Strain Rate, *BC* Breast Cancer, *RT* Radiotherapy; *p*-values in bold are significant (<0.05)

#### Associations between GLS reduction > 10% and radiation and non-radiation factors

**Fig. 1:**
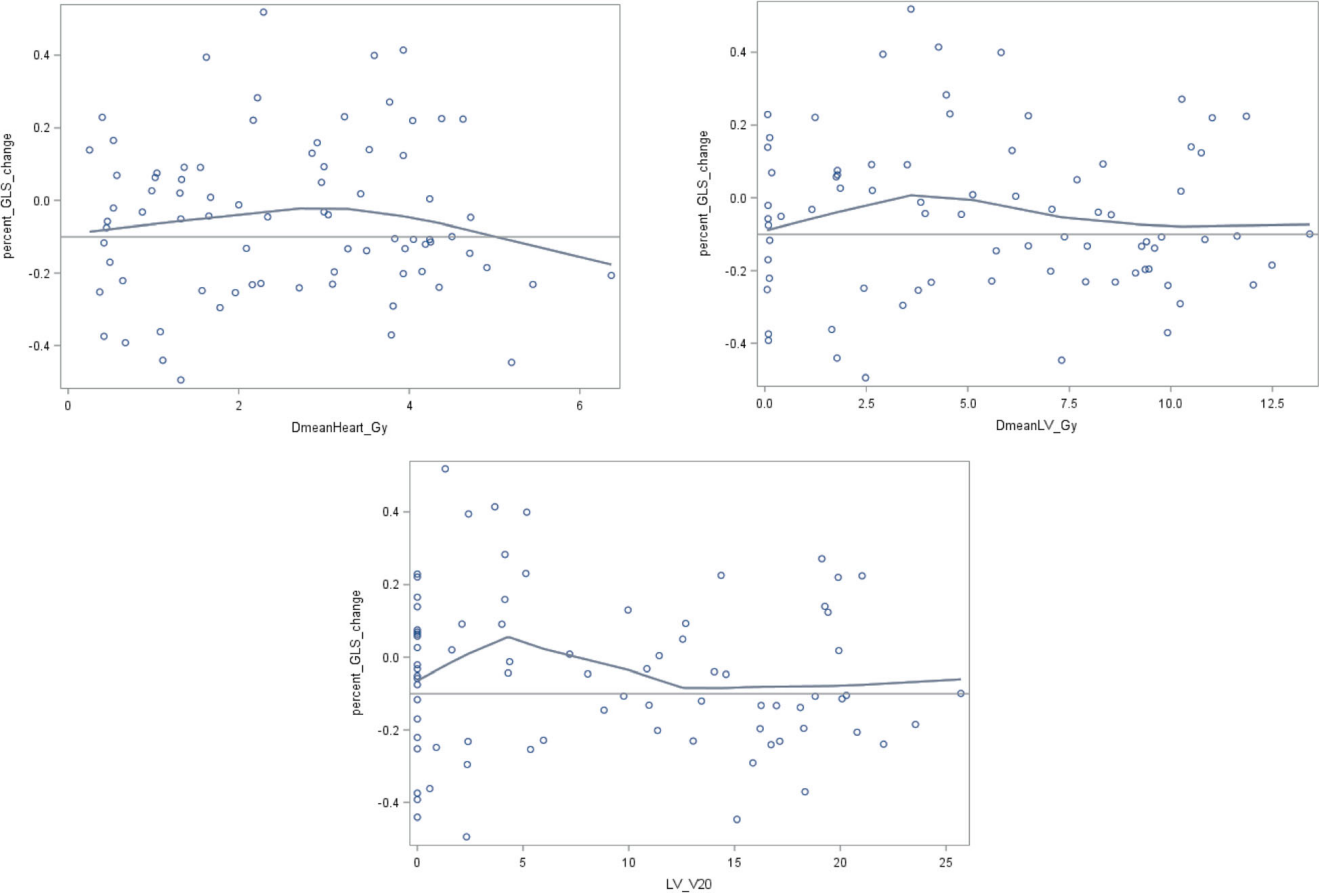
Continuous relationships between dosimetric variables (Dmean Heart, Dmean LV, LV V20) and GLS change: scatter plots with LOWESS curves

**Fig. 2:**
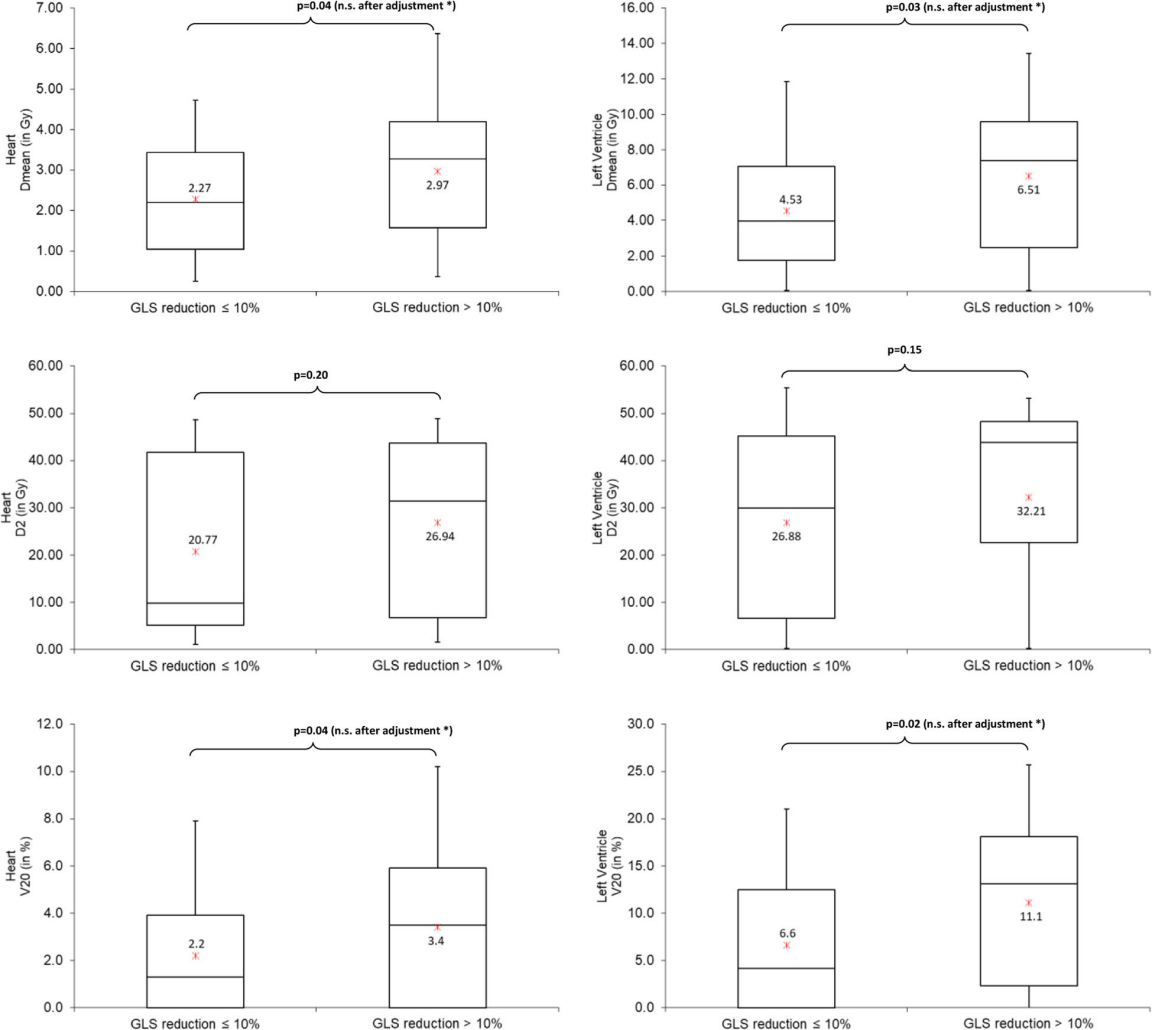
Boxplots for the distribution of heart and left ventricle (LV) doses and V20 according to the category without or with subclinical LV dysfunction (GLS reduction ≤10% and >10% respectively). * after Holm-Bonferroni methods for multiple testing, these *p*-values were no longer significant (n.s.)

**Fig. 3:**
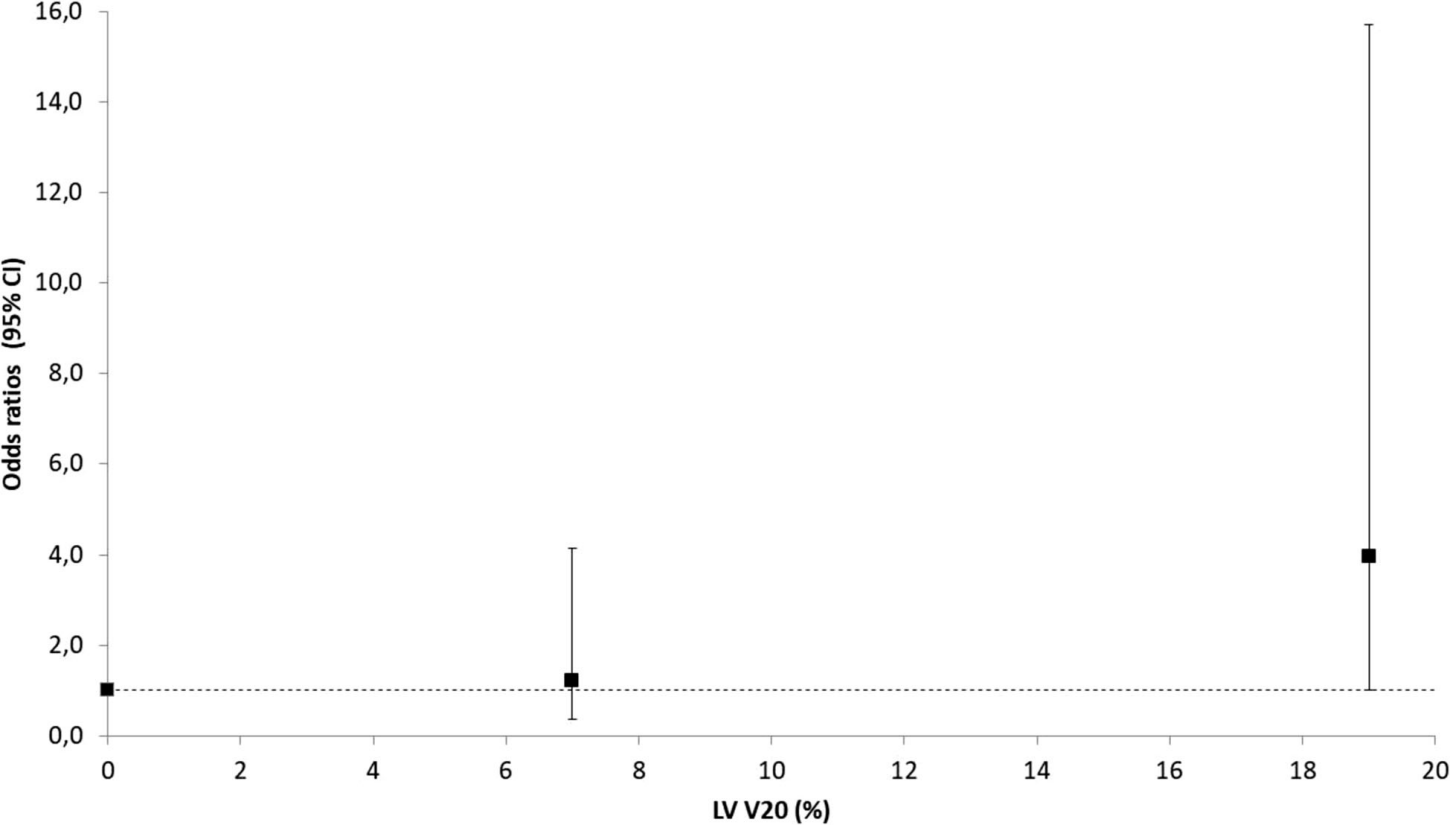
Exploratory analysis of the association between subclinical LV dysfunction defined as GLS reduction > 10% and V20 of the left ventricle (LV) divided in 3 categories: 0% (reference category, 21 patients, 7 patients with the event); 0% - 15% (mean = 7%, 35 patients, 13 patients with the event); >15% (mean = 19%, 23 patients, 17 patients with the event). Odds ratios are adjusted for BMI, hypercholesterolemia and endocrine therapy. For the highest category, OR = 3.97 [1.01 – 15.70], *p*=0.045

As illustrated in Fig. 1, no continuous association between dose measures and GLS change was observed with Spearman correlation coefficients between − 0.03 and − 0.10. The comparison of cardiac doses between patients with subclinical LV dysfunction (GLS reduction > 10%) and those without dysfunction (GLS reduction ≤10%) showed higher doses in heart or LV for patients with GLS reduction > 10%, in particular for LV V20 (11.1% vs. 6.6%) (Fig. 2). After Holm-Bonferroni method for multiple testing, none of these differences reached statistical significance. Among non-radiation factors associated with GLS reduction > 10%, BMI, hypercholesterolemia and endocrine therapy reached a *p*-value of less than 0.20 and were considered in further analysis (Table 4). The significant association with endocrine therapy (OR = 3.20 (1.02–1.30), *p* = 0.04), was in particular significant for patients treated with anti-aromatase (OR = 4.52 (1.32–15.53)). In univariate analysis, laterality of BC was not significantly associated with the event GLS reduction > 10% (*p* = 0.55), in contrast with mean dose to the heart and to the left ventricle (Table 5): Odds Ratio = 1.37, *p* = 0.04 for Dmean Heart; OR = 1.14, *p* = 0.03 for Dmean LV. Furthermore, an association was observed for LV V20 (OR = 1.08, *p* = 0.02). In multivariate analysis, none of these associations remained significant after adjustment for BMI, hypercholesterolemia and endocrine therapy. The LV V20 was the closest from statistical significance (OR = 1.05 [0.99–1.12], *p* = 0.12). In further exploratory analysis of the LV V20, patients were grouped according to whether the LV V20 was 0%, less than 15%, or 15% or more. There was a 4-fold increase in the adjusted odds ratio for subclinical LV dysfunction for the most exposed category, as compared with the 0% category (OR = 3.97 [1.01–15.74], *p* = 0.048) (Fig. 3).

**Table 4:** Non radiation factors associated with subclinical LV dysfunction (GLS reduction > 10%) after RT

	OR (95% CI)	*p*-value
Age (in years)	0.98 (0.93–1.04)	0.52
BMI (in Kg.m-^2^)	1.15 (1.02–1.30)	**0.02**^**a**^
Smoking		
Former vs. No	0.59 (0.20–1.68)	0.31
Current vs No	1.98 (0.57–6.91)	0.28
Hypertension	1.03 (0.37–2.89)	0.95
Diabetes	1.76 (0.28–11.19)	0.55
Hypercholesterolemia	1.95 (0.74–5.15)	**0.18**^**a**^
Endocrine therapy	3.20 (1.02–10.10)	**0.04**^**a**^
Anti-aromatase	4.52 (1.32–15.53)	**0.02**
Tamoxifen	2.05 (0.57–7.41)	0.27
RT protocol (hypofractionnated vs. standard)	0.91 (0.33–2.51)	0.84

*BMI* Body Mass Index, ^a^variables with *p*-value < 0.20 are considered for adjustment in multivariate analysis for the relationship between cardiac exposure and GLS reduction > 10%

**Table 5:** Associations between cardiac radiation doses and subclinical LV dysfunction (GLS reduction > 10%) after RT

	Univariate analysis		Multivariate analysis^a^	
	OR (95% CI)	*p*-value	OR (95% CI)	*p*-value
Laterality of BC (left vs. right)	1.41 (0.45–4.42)	0.55	–	–
Heart				
Dmean (Gy)	1.37 (1.01–1.86)	**0.04**	1.21 (0.87–1.71)	0.26
D2 (Gy)	1.02 (0.99–1.05)	0.13	–	–
V20 (%)	1.20 (1.01–1.43)	**0.04**	1.13 (0.93–1.36)	0.23
Left Ventricle				
Dmean (Gy)	1.14 (1.01–1.28)	**0.03**	1.09 (0.96–1.25)	0.17
D2 (Gy)	1.02 (0.99–1.04)	0.22	–	–
V20 (%)	1.08 (1.01–1.14)	**0.02**	1.05 (0.99–1.12)	0.12

^a^Adjusted for BMI, hypercholesterolemia and endocrine therapy; *p*-values in bold are significant (<0.05)

## Discussion

In this prospective study, we evaluated the association between cardiac exposure and subclinical LV dysfunction defined as GLS reduction > 10% from baseline before RT to 6 months after RT in BC patients treated with 3D-CRT without chemotherapy. The associations with mean doses to the heart and the LV as well as the heart and LV V20 were significant in univariate analysis. In multivariate analysis, these associations were no longer significant after adjustment for non-radiation factors including BMI, hypercholesterolemia and endocrine therapy. Further exploratory analysis allowed identifying a subgroup of patients (LV V20 > 15%) with a significant association with subclinical LV dysfunction which remained significant after adjustment.

Decrease in longitudinal strain was previously observed in left-sided BC patients with follow-up ranging from few days to 14 months after RT [11, 14, 15, 26] and mean relative decrease in longitudinal strain ranging from 5% to nearly 15% [12]. In our study, the mean decrease of GLS was 6% at 6 months after RT, in the range of previously observed decreases. The absence of significant decrease of GLS in right-sided BC patients was also previously observed in these studies even if the limited size of right-sided BC patients group could partly explain these non-significant results. Similarly to most other previous studies [12–15, 26], no significant decrease in LVEF was observed at RT + 6 months in our patients compared with pre-RT.

We considered a subclinical LV dysfunction defined as GLS reduction > 10% which has been considered clinically relevant [18] and which was also considered in other previous studies [17, 27]. This early index of cardiotoxicity was observed in 48% of our left-sided BC patients 6 months after RT, which is higher than the 28% observed elsewhere with a shorter follow-up limited to end of RT [14]. Such subclinical dysfunction may need longer follow-up to develop. No other study evaluated the frequency of this subclinical event as they mainly considered the GLS as a continuous variable which may limit the clinical implication and applications.

Even if all previous studies concluded that longitudinal strain was decreased after RT for left-sided BC patients and not for right-sided BC patients, little was known on the association between cardiac doses and decrease in longitudinal strain. A modest correlation was observed between GLS reduction 6 weeks after RT and mean heart dose or V30 (*R* = 0.35, *R* = 0.22) [26, 27], but no difference in radiation dose between the group with or without a > 10% reduction in GLS was observed. That could be explained by the limited number of patients and also the potential contribution of factors apart from radiation dose [16]. With a very short follow-up (end of RT), another study did not find an association between mean heart dose and the mean GLS reduction [14]. In our study, we observed significant associations between cardiac doses and subclinical LV dysfunction defined as GLS reduction > 10%. However, these associations were no longer significant after adjustment for non-radiation factor also associated with subclinical LV dysfunction including BMI, hypercholesterolemia and endocrine therapy. It was interesting to note that LV exposure, in particular V20 of the LV, was associated with subclinical LV dysfunction. As exploratory analysis, we identified a subgroup of patients with LV V20 > 15% and there was for this group a 4-fold increase in the adjusted odds ratio for subclinical LV dysfunction, as compared with the 0% category. This may be an indication for future research investigating strain evolution post-RT and subclinical LV dysfunction: instead of whole heart doses, analysis of LV exposure may provide better information to understand the association with RT. In a previous work published by Van Den Bogaard et al. [5], an association between the volume of the left ventricle receiving 5 Gy (LV-V5) and cumulative incidence of acute coronary event (ACE) was observed. Their analysis showed that LV-V5 was the most important prognostic dose-volume parameter. In our study, LV-V5 was significantly associated with GLS reduction > 10% in univariate analysis (OR = 1.04 [1.00–1.08], *p* = 0.04) but was no longer significant in multivariate analysis (OR = 1.03 [0.99–1.07], *p* = 0.21).

Contribution of factors apart from radiation dose on the risk of long term cardiac disease, such as age, hypertension, diabetes or preexisting cardiac diseases was previously observed [3] as they had an additive effect on the risk of cardiac disease. At the scale of subclinical LV dysfunction, quantified by longitudinal strain, it was also important to consider their contribution on the associations. Among the different factors that we considered, endocrine therapy was associated with subclinical LV dysfunction (OR = 3.20, 95% CI (1.02–10.10)), in particular for aromatase inhibitors (OR = 4.25, 95% CI (1.32–15.53)) which are known risk factors for cardiovascular disease [28]. An independent association between a reduction in GLS and the use of aromatase inhibitors was also previously observed [14]. The BMI was also important to consider in multivariate analysis, as it was a confounding factor: associated with both the cardiac event and the dose. We observed that patients with higher BMI had higher cardiac doses (Heart Dmean = 2.1 Gy for patient with BMI < 25 kg/m^2^ vs. 3.3 Gy for patients with BMI > 25 kg/m^2^, *p* < 0.01), as previously observed [29]. Last, the cardiotoxicity of chemotherapy, such as anthracyclines or trastuzumab, are known to alter the longitudinal strain [30, 31]. A strength of our study was to include chemotherapy-naïve patients, which allowed a precise evaluation of the association with radiation exposure without confounding due to chemotherapy.

Some studies investigated more precisely the longitudinal strain changes based on the segmental evaluation within the LV [32]. Lo et al. detected dose-related regional myocardial dysfunction in the acute phase after RT with the greatest reduction in the apical part of the LV, which received the highest radiation dose [15]. In the Erven’s study [11], changes were more pronounced in the LV wall receiving the highest RT dose (anterior wall). Heterogeneity of cardiac exposure [20] may be considered for precise evaluation of cardiotoxicity with the assessment of doses to cardiac substructures such as LV or LAD. The LV segmentations and assignment of these segments to coronary arterial territories are still not sharply defined and confusing [32]. However, we are now collecting strain segmental values for each echocardiography of our patients and will further analyze this data according to the precise individual dose evaluation of each coronary artery [20]. On the other side, the strain is not uniform over the LV. The LV wall is not homogenous and comprises an endocardial, a mid-myocardial, and an epicardial layer [25]. Recent 2DSTE software allows separate evaluation of myocardial layers deformation [33]. Our measurements of longitudinal strain focused on individual evaluation of midwall deformations. Our results at baseline (GLS = − 16.1 ± 2.7%) is consistent with the GLS observed in previous study [34]. Other studies averaged the three layers of GLS [11, 26], but we chose to focus here on the middle layer considering that this GLS would be a good indicator of the association with radiation exposure in the LV wall in addition to the fact that it has been shown in several studies to be robust and reproducible [24]. Further analysis detailing the three layers are in progress and may allow refining our results as previously observed for chemotherapy [35].

Research on radiation-induced changes in LV-function and association with cardiac exposure in patients treated with RT is not only relevant for BC patients. Patients treated for other organ with relevant heart doses like esophagus cancer, lung cancer or Hodgkin’s lymphoma are also of concerns. Some studies showed that, similarly to BC patients, these patients could receive high doses to the heart and its substructures [36] and GLS could be decreased after lung RT [37]. It is thus important to further develop studies, not only for BC patients, but also for other patients with thoracic RT.

### Limitations

Even if we could observe associations between cardiac doses and the risk of subclinical LV dysfunction, our study showed the importance to consider also non-radiation factors such as BMI or endocrine therapy in the investigation of these association with doses as the results didn’t remain significant after adjustment. Our study is one of the largest ever published in this research area. However, with 79 patients, the size of the population involved a limited statistical power for odds ratios sub-analyses which may partly explain the absence of significant results in multivariate analysis. A large multicenter European study (MEDIRAD EARLY-HEART study) is ongoing and plan to include 250 patients which should provide results without this limitation [38]. Some alternatives to cardiac echocardiography for assessment of early cardiac damage exist, such as myocardial scintigraphy or cardiac magnetic resonance imaging. Myocardial scintigraphy is an important noninvasive method in the evaluation of patients with suspected coronary artery disease due to its high diagnostic accuracy, as well as being able to define the extent, severity and location of myocardial perfusion abnormalities. It is used for detecting ischemia in symptomatic patients, but its use in asymptomatic ones, like our BC patients, is less clear, which conducted us not to consider this examination in our cohort. However cardiac magnetic resonance imaging for assessment of the function and structure of the cardiovascular system is very promising and will be further investigated in MEDIRAD EARLY-HEART study [38]. The subclinical LV dysfunction defined as a GLS reduction > 10% is an indication of a beginning effect of radiation on LV function which corresponded to a secondary endpoint of BACCARAT study [21].

## Conclusions

This is the first study to investigate the associations between BC RT-induced cardiac doses and subclinical LV dysfunction defined as a GLS reduction > 10%, 6 months after RT. This 6-month follow-up analysis indicated a significant association with mean heart dose, V20 of the heart, mean LV dose and V20 of the LV in univariate analysis. However, they didn’t remain significant in multivariate analysis in particular after adjustment for endocrine therapy. An exploratory analysis in our study allowed identifying a subgroup of patients (LV V20 > 15%) for whom a significant association with subclinical LV dysfunction was observed. LV dosimetry may be promising to identify high-risk subpopulations. Larger and longer follow-up studies are required to further investigate these associations.

## Data Availability

The datasets used and/or analyzed during the current study are available from the corresponding author on reasonable request.

## Abbreviations

3D-CRT: 3 Dimensional Conformal Radiotherapy
BACCARAT: Breast Cancer and Cardiotoxicity Induced by Radiotherapy
BC: Breast Cancer
BMI: Body Mass Index
CT: Computed Tomography
DVH: Dose Volume Histogram
GLS: Global Longitudinal Strain
GLSR: Global Longitudinal Strain Rate
LS: Longitudinal Strain
LV: Left Ventricle
LVEF: Left Ventricle Ejection Fraction
MHD: Mean Heart Dose
RT: Radiotherapy
TPS: Treatment Planning System
